# Supplementary material for: Pyruvate Dehydrogenase Kinase 1 inhibition mediated oxidative phosphorylation enhancement in cartilage promotes osteoarthritis progression
Source: BMC Musculoskelet Disord. 2023 Jul 20;24:597. doi: 10.1186/s12891-023-06585-6 (PMC10357736; doi:10.1186/s12891-023-06585-6)
Supplement: Supplementary file 1 — Additional file 1. [file 12891_2023_6585_MOESM1_ESM.zip › KEGG.Gsea.1653622432464/KEGG_PROGESTERONE_MEDIATED_OOCYTE_MATURATION.html]

Details for gene set KEGG\_PROGESTERONE\_MEDIATED\_OOCYTE\_MATURATION[GSEA]

|  || Dataset | 55457\_collapsed\_to\_symbols.cls.cls #OA\_versus\_normal.cls.cls #OA\_versus\_normal\_repos |
| Phenotype | cls.cls#OA\_versus\_normal\_repos |
| Upregulated in class | normal |
| GeneSet | KEGG\_PROGESTERONE\_MEDIATED\_OOCYTE\_MATURATION |
| Enrichment Score (ES) | -0.2646436 |
| Normalized Enrichment Score (NES) | -1.0897456 |
| Nominal p-value | 0.31484503 |
| FDR q-value | 0.52311754 |
| FWER p-Value | 1.0 |
Table: GSEA Results Summary

  

Fig 1: Enrichment plot: KEGG\_PROGESTERONE\_MEDIATED\_OOCYTE\_MATURATION      
 Profile of the Running ES Score & Positions of GeneSet Members on the Rank Ordered List

  

| SYMBOL | TITLE | RANK IN GENE LIST | RANK METRIC SCORE | RUNNING ES | CORE ENRICHMENT || 1 | MAPK9 | mitogen-activated protein kinase 9 [Source:HGNC Symbol;Acc:HGNC:6886] | 721 | 0.588 | -0.0300 | No |
| 2 | MAPK3 | mitogen-activated protein kinase 3 [Source:HGNC Symbol;Acc:HGNC:6877] | 757 | 0.579 | -0.0069 | No |
| 3 | PRKACB | protein kinase cAMP-activated catalytic subunit beta [Source:HGNC Symbol;Acc:HGNC:9381] | 771 | 0.576 | 0.0178 | No |
| 4 | ANAPC13 | anaphase promoting complex subunit 13 [Source:HGNC Symbol;Acc:HGNC:24540] | 1116 | 0.493 | 0.0129 | No |
| 5 | AKT1 | AKT serine/threonine kinase 1 [Source:HGNC Symbol;Acc:HGNC:391] | 1192 | 0.478 | 0.0284 | No |
| 6 | ADCY7 | adenylate cyclase 7 [Source:HGNC Symbol;Acc:HGNC:238] | 1408 | 0.442 | 0.0314 | No |
| 7 | MAD2L1 | mitotic arrest deficient 2 like 1 [Source:HGNC Symbol;Acc:HGNC:6763] | 1516 | 0.427 | 0.0420 | No |
| 8 | ADCY1 | adenylate cyclase 1 [Source:HGNC Symbol;Acc:HGNC:232] | 1589 | 0.415 | 0.0549 | No |
| 9 | PIK3CB | "phosphatidylinositol-4,5-bisphosphate 3-kinase catalytic subunit beta [Source:HGNC Symbol;Acc:HGNC:8976]" | 2186 | 0.340 | 0.0236 | No |
| 10 | IGF1 | insulin like growth factor 1 [Source:HGNC Symbol;Acc:HGNC:5464] | 2286 | 0.328 | 0.0306 | No |
| 11 | MAPK14 | mitogen-activated protein kinase 14 [Source:HGNC Symbol;Acc:HGNC:6876] | 2352 | 0.320 | 0.0398 | No |
| 12 | CCNB1 | cyclin B1 [Source:HGNC Symbol;Acc:HGNC:1579] | 2359 | 0.319 | 0.0535 | No |
| 13 | BUB1 | BUB1 mitotic checkpoint serine/threonine kinase [Source:HGNC Symbol;Acc:HGNC:1148] | 2388 | 0.315 | 0.0654 | No |
| 14 | MAPK13 | mitogen-activated protein kinase 13 [Source:HGNC Symbol;Acc:HGNC:6875] | 2544 | 0.298 | 0.0666 | No |
| 15 | RPS6KA1 | ribosomal protein S6 kinase A1 [Source:HGNC Symbol;Acc:HGNC:10430] | 3389 | 0.213 | 0.0103 | No |
| 16 | PLK1 | polo like kinase 1 [Source:HGNC Symbol;Acc:HGNC:9077] | 3556 | 0.198 | 0.0062 | No |
| 17 | GNAI3 | G protein subunit alpha i3 [Source:HGNC Symbol;Acc:HGNC:4387] | 3694 | 0.186 | 0.0038 | No |
| 18 | CDC23 | cell division cycle 23 [Source:HGNC Symbol;Acc:HGNC:1724] | 3729 | 0.183 | 0.0093 | No |
| 19 | CDK1 | cyclin dependent kinase 1 [Source:HGNC Symbol;Acc:HGNC:1722] | 3744 | 0.182 | 0.0163 | No |
| 20 | INS | insulin [Source:HGNC Symbol;Acc:HGNC:6081] | 3885 | 0.166 | 0.0128 | No |
| 21 | CDC16 | cell division cycle 16 [Source:HGNC Symbol;Acc:HGNC:1720] | 4566 | 0.108 | -0.0354 | No |
| 22 | ADCY9 | adenylate cyclase 9 [Source:HGNC Symbol;Acc:HGNC:240] | 5191 | 0.057 | -0.0815 | No |
| 23 | ANAPC10 | anaphase promoting complex subunit 10 [Source:HGNC Symbol;Acc:HGNC:24077] | 5424 | 0.039 | -0.0979 | No |
| 24 | CDC25A | cell division cycle 25A [Source:HGNC Symbol;Acc:HGNC:1725] | 5666 | 0.018 | -0.1159 | No |
| 25 | PDE3B | phosphodiesterase 3B [Source:HGNC Symbol;Acc:HGNC:8779] | 6077 | -0.010 | -0.1474 | No |
| 26 | RAF1 | "Raf-1 proto-oncogene, serine/threonine kinase [Source:HGNC Symbol;Acc:HGNC:9829]" | 6187 | -0.019 | -0.1550 | No |
| 27 | CPEB1 | cytoplasmic polyadenylation element binding protein 1 [Source:HGNC Symbol;Acc:HGNC:21744] | 6435 | -0.038 | -0.1726 | No |
| 28 | ADCY8 | adenylate cyclase 8 [Source:HGNC Symbol;Acc:HGNC:239] | 6646 | -0.053 | -0.1866 | No |
| 29 | ANAPC5 | anaphase promoting complex subunit 5 [Source:HGNC Symbol;Acc:HGNC:15713] | 6751 | -0.059 | -0.1921 | No |
| 30 | ANAPC2 | anaphase promoting complex subunit 2 [Source:HGNC Symbol;Acc:HGNC:19989] | 6787 | -0.062 | -0.1921 | No |
| 31 | MAPK1 | mitogen-activated protein kinase 1 [Source:HGNC Symbol;Acc:HGNC:6871] | 6853 | -0.068 | -0.1941 | No |
| 32 | RPS6KA3 | ribosomal protein S6 kinase A3 [Source:HGNC Symbol;Acc:HGNC:10432] | 7092 | -0.087 | -0.2088 | No |
| 33 | PIK3CD | "phosphatidylinositol-4,5-bisphosphate 3-kinase catalytic subunit delta [Source:HGNC Symbol;Acc:HGNC:8977]" | 7405 | -0.111 | -0.2281 | No |
| 34 | PIK3CA | "phosphatidylinositol-4,5-bisphosphate 3-kinase catalytic subunit alpha [Source:HGNC Symbol;Acc:HGNC:8975]" | 7733 | -0.136 | -0.2476 | No |
| 35 | PIK3CG | "phosphatidylinositol-4,5-bisphosphate 3-kinase catalytic subunit gamma [Source:HGNC Symbol;Acc:HGNC:8978]" | 7953 | -0.152 | -0.2579 | Yes |
| 36 | AKT3 | AKT serine/threonine kinase 3 [Source:HGNC Symbol;Acc:HGNC:393] | 8008 | -0.156 | -0.2551 | Yes |
| 37 | CCNA2 | cyclin A2 [Source:HGNC Symbol;Acc:HGNC:1578] | 8047 | -0.159 | -0.2510 | Yes |
| 38 | RPS6KA6 | ribosomal protein S6 kinase A6 [Source:HGNC Symbol;Acc:HGNC:10435] | 8071 | -0.161 | -0.2457 | Yes |
| 39 | ARAF | "A-Raf proto-oncogene, serine/threonine kinase [Source:HGNC Symbol;Acc:HGNC:646]" | 8092 | -0.163 | -0.2400 | Yes |
| 40 | GNAI1 | G protein subunit alpha i1 [Source:HGNC Symbol;Acc:HGNC:4384] | 8110 | -0.164 | -0.2340 | Yes |
| 41 | MAPK12 | mitogen-activated protein kinase 12 [Source:HGNC Symbol;Acc:HGNC:6874] | 8161 | -0.168 | -0.2304 | Yes |
| 42 | BRAF | "B-Raf proto-oncogene, serine/threonine kinase [Source:HGNC Symbol;Acc:HGNC:1097]" | 8171 | -0.169 | -0.2236 | Yes |
| 43 | RPS6KA2 | ribosomal protein S6 kinase A2 [Source:HGNC Symbol;Acc:HGNC:10431] | 8397 | -0.185 | -0.2329 | Yes |
| 44 | ADCY3 | adenylate cyclase 3 [Source:HGNC Symbol;Acc:HGNC:234] | 8572 | -0.199 | -0.2375 | Yes |
| 45 | CCNA1 | cyclin A1 [Source:HGNC Symbol;Acc:HGNC:1577] | 8584 | -0.200 | -0.2295 | Yes |
| 46 | GNAI2 | G protein subunit alpha i2 [Source:HGNC Symbol;Acc:HGNC:4385] | 8686 | -0.208 | -0.2281 | Yes |
| 47 | PIK3R3 | phosphoinositide-3-kinase regulatory subunit 3 [Source:HGNC Symbol;Acc:HGNC:8981] | 8801 | -0.217 | -0.2273 | Yes |
| 48 | PIK3R5 | phosphoinositide-3-kinase regulatory subunit 5 [Source:HGNC Symbol;Acc:HGNC:30035] | 8804 | -0.217 | -0.2178 | Yes |
| 49 | CDC27 | cell division cycle 27 [Source:HGNC Symbol;Acc:HGNC:1728] | 8944 | -0.227 | -0.2185 | Yes |
| 50 | MAPK8 | mitogen-activated protein kinase 8 [Source:HGNC Symbol;Acc:HGNC:6881] | 8956 | -0.228 | -0.2092 | Yes |
| 51 | PGR | progesterone receptor [Source:HGNC Symbol;Acc:HGNC:8910] | 9048 | -0.235 | -0.2058 | Yes |
| 52 | MOS | "MOS proto-oncogene, serine/threonine kinase [Source:HGNC Symbol;Acc:HGNC:7199]" | 9138 | -0.243 | -0.2019 | Yes |
| 53 | CDC25C | cell division cycle 25C [Source:HGNC Symbol;Acc:HGNC:1727] | 9501 | -0.274 | -0.2179 | Yes |
| 54 | ANAPC1 | anaphase promoting complex subunit 1 [Source:HGNC Symbol;Acc:HGNC:19988] | 9630 | -0.286 | -0.2151 | Yes |
| 55 | MAPK10 | mitogen-activated protein kinase 10 [Source:HGNC Symbol;Acc:HGNC:6872] | 9699 | -0.291 | -0.2074 | Yes |
| 56 | ADCY2 | adenylate cyclase 2 [Source:HGNC Symbol;Acc:HGNC:233] | 9715 | -0.293 | -0.1955 | Yes |
| 57 | FZR1 | fizzy and cell division cycle 20 related 1 [Source:HGNC Symbol;Acc:HGNC:24824] | 9996 | -0.321 | -0.2031 | Yes |
| 58 | HSP90AB1 | heat shock protein 90 alpha family class B member 1 [Source:HGNC Symbol;Acc:HGNC:5258] | 10060 | -0.327 | -0.1934 | Yes |
| 59 | MAP2K1 | mitogen-activated protein kinase kinase 1 [Source:HGNC Symbol;Acc:HGNC:6840] | 10197 | -0.340 | -0.1888 | Yes |
| 60 | CDC25B | cell division cycle 25B [Source:HGNC Symbol;Acc:HGNC:1726] | 10206 | -0.341 | -0.1742 | Yes |
| 61 | PRKACG | protein kinase cAMP-activated catalytic subunit gamma [Source:HGNC Symbol;Acc:HGNC:9382] | 10466 | -0.370 | -0.1779 | Yes |
| 62 | KRAS | "KRAS proto-oncogene, GTPase [Source:HGNC Symbol;Acc:HGNC:6407]" | 10544 | -0.377 | -0.1671 | Yes |
| 63 | PRKACA | protein kinase cAMP-activated catalytic subunit alpha [Source:HGNC Symbol;Acc:HGNC:9380] | 10611 | -0.384 | -0.1551 | Yes |
| 64 | CCNB2 | cyclin B2 [Source:HGNC Symbol;Acc:HGNC:1580] | 10639 | -0.387 | -0.1400 | Yes |
| 65 | PIK3R2 | phosphoinositide-3-kinase regulatory subunit 2 [Source:HGNC Symbol;Acc:HGNC:8980] | 10956 | -0.425 | -0.1456 | Yes |
| 66 | CDK2 | cyclin dependent kinase 2 [Source:HGNC Symbol;Acc:HGNC:1771] | 11069 | -0.441 | -0.1347 | Yes |
| 67 | PIK3R1 | phosphoinositide-3-kinase regulatory subunit 1 [Source:HGNC Symbol;Acc:HGNC:8979] | 11099 | -0.443 | -0.1172 | Yes |
| 68 | PKMYT1 | "protein kinase, membrane associated tyrosine/threonine 1 [Source:HGNC Symbol;Acc:HGNC:29650]" | 11403 | -0.487 | -0.1191 | Yes |
| 69 | MAPK11 | mitogen-activated protein kinase 11 [Source:HGNC Symbol;Acc:HGNC:6873] | 11501 | -0.501 | -0.1043 | Yes |
| 70 | PDE3A | phosphodiesterase 3A [Source:HGNC Symbol;Acc:HGNC:8778] | 11546 | -0.507 | -0.0852 | Yes |
| 71 | PRKX | protein kinase X-linked [Source:HGNC Symbol;Acc:HGNC:9441] | 11634 | -0.522 | -0.0686 | Yes |
| 72 | ADCY6 | adenylate cyclase 6 [Source:HGNC Symbol;Acc:HGNC:237] | 12005 | -0.594 | -0.0710 | Yes |
| 73 | IGF1R | insulin like growth factor 1 receptor [Source:HGNC Symbol;Acc:HGNC:5465] | 12689 | -0.855 | -0.0861 | Yes |
| 74 | HSP90AA1 | heat shock protein 90 alpha family class A member 1 [Source:HGNC Symbol;Acc:HGNC:5253] | 12730 | -0.893 | -0.0494 | Yes |
| 75 | AKT2 | AKT serine/threonine kinase 2 [Source:HGNC Symbol;Acc:HGNC:392] | 12896 | -1.404 | 0.0003 | Yes |
Table: GSEA details [plain text format]

  

Fig 2: KEGG\_PROGESTERONE\_MEDIATED\_OOCYTE\_MATURATION      
 Blue-Pink O' Gram in the Space of the Analyzed GeneSet

  

Fig 3: KEGG\_PROGESTERONE\_MEDIATED\_OOCYTE\_MATURATION: Random ES distribution      
 Gene set null distribution of ES for **KEGG\_PROGESTERONE\_MEDIATED\_OOCYTE\_MATURATION**
